# Clinical clusters during acute illness predict long-term mortality in older patients

**DOI:** 10.1186/s12916-025-04500-5

**Published:** 2025-12-29

**Authors:** A. Tsui, P. Hogan, H. Cheston, O. Dunne, D. Gardner, A. McWhirter, L. M. Allan, S. Richardson, P. Nachev, D. Davis

**Affiliations:** 1 Institute of Health Informatics, University College London (UCL), 222 Euston Road, London, NW12DA UK; 2 Central and North West London NHS Trust, London, UK; 3 Univerisity College London Hospitals NHS Trust, 250 Euston Road, London, NW1 2PB UK; 4 Translational and Clinical Research Institute, Faculty of Medical Sciences, Newcastle University, Newcastle, UK; 5 Wellcome Centre for Human Neuroimaging, UCL, London, UK; 6 UCL Queen Square Institute of Neurology, UCL, London, UK; 7 College of Medicine and Health, South Cloisters, University of Exeter, Exeter, EX1 2LU UK

**Keywords:** Subtypes, Clusters, Prediction, Frailty, Geriatrics, Acute illness, High-dimensional, Multi-modal modelling

## Abstract

**Background:**

Defining acute illness decompensation as a single entity limits individualisation of treatments for older patients. Multi-modal and high-dimensional data offer opportunities to derive quantified clusters with clinically meaningful outcomes. We tested the hypothesis that cluster-driven and high-dimensional predictors can be constructed with sufficient fidelity for clinical deployment, concurrently highlighting mechanistic insights into pathophysiological substrates of acute illness decompensation, including where this affected the brain.

**Methods:**

Two independent prospective cohort studies, DELPHIC and DECIDE, were harmonised and utilised as train and test partitions, contributing 209 and 205 unique first-participant acute admission episodes respectively. Baseline and acute illness variables were projected using T-stochastic neighbour embedding onto a two-dimensional manifold and agglomerative hierarchical clustering designated distance-defined subtypes. Predictive performances of clusters and full models were compared for brain decompensation within admission and 2-year mortality. SHapley Additive exPlanations (SHAPs) quantified directional contributions of inputs towards high-dimensional model performances.

**Results:**

Three broad clinical subtypes were identified in older people during decompensation, with similar contributions from baseline and acute illness variables. From baseline to cluster-driven, and then high-dimensional prediction models for brain decompensation in admission, the test area under receiver operating characteristic curve (AUROC) improved from 0.563 to 0.641 and 0.797 respectively. Sleep–wake cycle disturbance was the most important predictor of delirium in admission, while physiological fluctuations within an admission episode, in particular from cognitive domains, were significant predictors of long-term mortality after acute admission.

**Conclusions:**

Robust, generalisable clusters with clinical utility are discernable for older patients during acute illness. Our results demonstrate proof of concept for a longitudinal approach towards defining and modelling acute illness. We illustrate the potential to maximally predict adverse outcomes with high-dimensionality and multi-modality, and highlight the importance of sleep–wake cycle disturbances as a future target in studies of delirium neuropathophysiology.

**Supplementary Information:**

The online version contains supplementary material available at 10.1186/s12916-025-04500-5.

## Background

When older patients are hospitalised with acute illness, a wide range of factors can impact clinical outcomes. This population is profoundly heterogeneous: there is large variation in how multimorbidity and functional impairments chronically accumulate, interact with socioeconomic factors over the life course [1], juxtapose with a myriad of acute aetiologies, and result in divergent patterns of decompensation to high-order functions such as sustained attention (delirium) or neuromuscular control of balance and gait (falls). In addition, presenting phenotypes are dynamic over the short and long term, where there are differences in the pattern and severities of both the baseline (cognitive and physical function) and acute components (inattention, altered arousal, sleep–wake disturbance, other neuropsychiatric and motor deficits). Disaggregating these clinical variations requires detailed prospective studies capturing individuals before, during and after acute illness [2], and studies mapping this in population samples have shown divergent outcomes [3–5]. Data-driven delirium diagnoses also offer an opportunity to measure the effectiveness of validated screening instruments such as the 4AT in routine clinical practice [6].

While acute illness is recognised to result in multiple adverse long-term outcomes in the frailest cohorts [7, 8], more granular definitions of acute decompensation are vital at both population and individual levels. Such definitions would enable the identification of robust and reproducible clusters with prognostic implications, supporting stratified approaches to management and inform the development of future therapeutics. At an individual level, while complex, high-dimensional models may produce the greatest predictive performances, particularly as data resolution and number of modalities increase, such algorithms have substantial computational demands which limits their scope for practical implementation. A dimensionality-reductive approach is an attractive compromise: algorithms using such techniques might yield marginally reduced yet comparable performance while offering sufficient scalability to be clinically and operationally effective.

The primary goal of this study was to use two comparable prospective cohorts to define longitudinal clinical subtypes within baseline features and subsequent acute illness decompensation. Previous models of acute illness in older patients have had poor or uncertain generalisability, most commonly due to a lack of external validation [9, 10]. With the advantage of data from longitudinal population studies of older people in acute illness, contemporaneously recruited, the Delirium and Population Health Informatics Cohort (DELPHIC) [4, 11] and Delirium and Cognitive Impact in Dementia (DECIDE) [3] studies offer the first externally validated data-driven longitudinal subtypes of acute illness in older people in inpatient settings. Second, we aimed to link identified subtypes with clinical outcomes. Third, we compared predictive performances of using cluster-driven and high-dimensional models, quantifying if the most accurate models demonstrated sufficient fidelity to deploy in clinical settings. Last, we considered mechanistic insights into pathological substrates for brain decompensation, highlighting potential targets for future studies.

## Methods

### Cohorts

We used data from two independent British population cohorts, DELPHIC and DECIDE. Both have been described in detail [12, 13]. DELPHIC was a longitudinal prospective sample, recruiting 1511 community-based participants aged ≥ 70 between 2018 and 2020 from the London Borough of Camden, with baseline assessments at recruitment, median follow-up at 3 years and daily assessments during any incident hospitalisation to University College London and Royal Free hospitals. DECIDE was a prospective sample nested within the Cognitive Function and Ageing Study II (CFAS-II), in which 1751 eligible participants ≥ 65 years were also assessed during each hospital admission to the Royal Victoria Infirmary or Freeman hospitals, Newcastle, between 5th January 2016 and 5th January 2017, followed by a repeat assessment 12 months after their most recent hospital admission. At follow-up for both cohorts, the same cognitive and functional assessments were repeated, as well as ascertainment of active medical diagnoses, to quantify changes during the study in relation to acute illness episodes.

### Data harmonisation

Variable groups common to both studies, operationalised as continuous features and suitable for harmonisation, were baseline cognition, baseline function (Barthel Index), clinical frailty score, educational attainment (years of education), acute biochemical markers, physiological markers (NEWS2 components), functional impairments (Hierarchical Assessment of Balance and Mobility—HABAM) [14], arousal deficits (Observational Scale of Level of Arousal—OSLA) [15] and delirium severity (Memorial Delirium Assessment Scale—MDAS) [16]. For the specific cognitive instruments, in DELPHIC, baseline cognition was based on TICS-m [17]; DECIDE used the mini-mental state examination (MMSE) and Cambridge Cognitive Examination (CAMCOG) [18]. Our primary outcomes were binary: cognitive decompensation (delirium within the admission episode) and 2-year mortality. We harmonised a scaled score for *orientation* out of 3 based on itemised descriptions for each scale. For variables collected over multiple days of a hospital admission (e.g. MDAS items, heart rate, repeat blood tests), we summarised data across the admission episode using minimum, maximum, mean and standard deviation to offer a representative measure of cognition, function, physiological and biochemical perturbations. Prescribed medications were translated using World Health Organisation (WHO) Anatomical Therapeutic Chemical (ATC) classification system, defined as levels 1 to 5; we used level 2 (Additional file 1: Supplementary Table 1). Missing data within each unique admission was filled forwards and backwards from any available data. Multiple imputation with chained equations was used for any continuous variables; missing drugs were filled as 0. Only data from the first chronological admission episode was used because within-person information from subsequent admissions would not be independent for test/train purposes.

### T-stochastic neighbour embedding (TSNE) and hierarchical clustering

We constructed a matrix of Gower distances between each non-Bernoulli variable as well as calculating an alternative using standardisation and centring, with the assumption of all data being linear and continuous. Projecting these in two-dimensional spaces as a first step in visualising potential clusters, we used T-stochastic neighbour embedding (TSNE) [19], with interpolation points-based modular Python implementation (openTSNE) to define an approximate function and allowing addition of new data points onto an existing embedding [20], to compare how 2D projections from training and test datasets were distributed and that any potential clusters were not a consequence of data source alone.

The first two-dimensional manifold was constructed using DELPHIC data only (perplexity: 20; metric: Euclidean); DECIDE data were added on to the same function and visually examined for similarity in space in a two-dimensional scatterplot (Additional file 2: Supplementary Fig. 1).

Clusters were delineated hierarchically using Euclidean distances between continuous variables with a “ward” definition of affinity, in which similarity was defined by the average squared distance between all pairs of datapoints. A horizontally drawn line across a dendrogram of hierarchical distances at the point of greatest vertical separation guided the optimal number of clusters, followed by manual inspection to ensure no cluster was composed of small numbers of data points. Interrogating feature contributions towards each cluster designation, predictors were univariately regressed against the cluster class. Similarly, for medication prescribed, the percentage of drugs prescribed for patients within each cluster was calculated.

### Predictive modelling

Survival up to 730 days (2 years) was plotted in a Kaplan–Meier graph by cohort and cluster designation. Difference in survival trajectories between clusters were analysed using log-rank test with *cohort* as an interaction term.

We estimated predictive models of hierarchically increasing complexity, using first baseline variables of age and sex only, then cluster class and finally, full individual baseline and acute admission features. Targets were acute cognitive decompensation (delirium) and 2-year mortality. XGBoost was selected for high-dimensional modelling due to its flexibility, ability to use mixed data, ease of hyperparameter optimisation and non-linear efficiency. In all models, data from DELPHIC were used for training and DECIDE for independent testing. Each partition contained only unique patients, with the first chronological admission episode selected for each patient. Ten-fold cross-validation was performed in the training DELPHIC dataset to quantify model performance variations and optimised with area under the receiver operating characteristic curve (AUROC) as the evaluation metric to minimise over/underfitting. For the cognitive decompensation (delirium) model, AUROC was selected as the evaluation metric instead of precision recall receiver operating characteristic curve (PRAUC) despite an outcome imbalance, as it is clinically equally important to predict the absence of delirium as it is to predict the future presence of delirium. A calibration curve was also plotted to demonstrate predicted performance across a range of observed values. Manual grid searching determined the most optimal hyperparameters (number of estimators, maximum depth, minimum child weight, learning rate, gamma, subsample, column sample by tree) (Additional file 3: Supplementary Table 2), which were subsequently deployed on the independent test dataset. The weighted directional contribution of each feature to the final model was determined using SHapley Additive exPlanations (SHAP) values, with the most important 20 features visually demonstrated on a directional heatmap.

## Results

### Cohort demographics

In DELPHIC, 209 participants were admitted over 372 number of admissions. Forty-five percent were diagnosed with delirium at any point during their included admission and 25% were no longer alive at 2 years after their first admission episode. In DECIDE, 205 number of participants were admitted over 318 number of admissions. Twenty-seven percent were diagnosed with delirium at any point and 22% were no longer alive at 2 years after their first admission episode. The mean ages for participants in DELPHIC and DECIDE were 80.6 and 81.6 years (*p* = 0.19) respectively. Baseline demographics were similar between both cohorts, although DELPHIC participants received more years of education (mean 11.3 compared with 10.1 years, *p* < 0.01). During acute illness, greater burden of delirium, arousal deficits and functional decline were demonstrated in DELPHIC patients, reflected in higher delirium incidence (DELPHIC 45%, DECIDE 26.8%, Table 1). However, acute illness severity as defined by NEWS2 was lower in DELPHIC (mean = 1.3) participants compared to DECIDE (mean = 5).

**Table 1:** Characteristics of included participants who have been admitted to hospital during study period, by cohort

	DELPHIC (*n* = 209)	DECIDE (*n* = 205)	*p*
Baseline MMSE (total/27)	23.3 (4.79)	23.74 (3.30)	0.23
Baseline fluency (words)	13.0 (7.00)	11.51 (5.46)	0.02
Baseline fluency (animals)	15.0 (7.78)	16.10 (5.62)	0.10
Age (years)	80.6 (6.32)	81.61 (8.65)	0.19
Sex (female)	54.07%	53.17%	0.20
Clinical frailty score (CFS)	4.13 (1.37)	4.22 (1.46)	0.52
Education (years)	11.3 (1.69)	10.1 (1.68)	< 0.01
Baseline Barthel (total)	89.4 (13.47)	90.59 (16.80)	0.44
Haemoglobin (g/L)	122.0 (5.46)	119.15 (5.05)	< 0.01
White blood cells (× 10^9^/L)	9.31 (1.58)	9.40 (1.70)	0.58
Platelets (× 10^9^/L)	265.8 (23.88)	261.29 (34.93)	0.12
Sodium (mmol/L)	137.6 (1.36)	138.71 (1.41)	< 0.01
Potassium (mmol/L)	4.32 (0.25)	4.24 (0.20)	< 0.01
Urea (mmol/L)	9.46 (9.05)	7.66 (2.10)	0.01
Creatinine (µmol/L)	93 (12.61)	98.95 (10.89)	< 0.01
C-reactive protein (g/L)	44.69 (39.53)	52.68 (37.06)	0.03
Albumin (mg/L)	38.86 (3.85)	38.54 (1.71)	0.28
Calcium (adjusted) (mmol/L)	2.19 (0.04)	2.38 (0.05)	< 0.01
MDAS total	9.07 (7.38)	4.29 (4.89)	< 0.01
OSLA total	2.78 (3.56)	0.45 (1.25)	< 0.01
HABAM total	21.6 (16.7)	41.8 (22.8)	< 0.01
NEWS total	1.29 (1.63)	5.04 (1.79)	< 0.01
Delirium during admission	44.97%	26.83%	< 0.01
Two-year mortality	24.88%	22.44%	< 0.01

Acute illness and biochemical data presented as mean (standard deviation) across first chronological admission episodes; *p* value refers to differences between two cohorts, using paired *t*-test for continuous measures and chi-squared for proportion variables

*MMSE *mini-mental state examination, *CFS *clinical frailty score, *CRP *C-reactive protein, *MDAS *Memorial Delirium Assessment Scale, *OSLA *Observational Scale of Level of Arousal, *HABAM *Hierarchical Assessment of Balance and Mobility, *NEWS *national early warning score 2

**Fig. 1:**
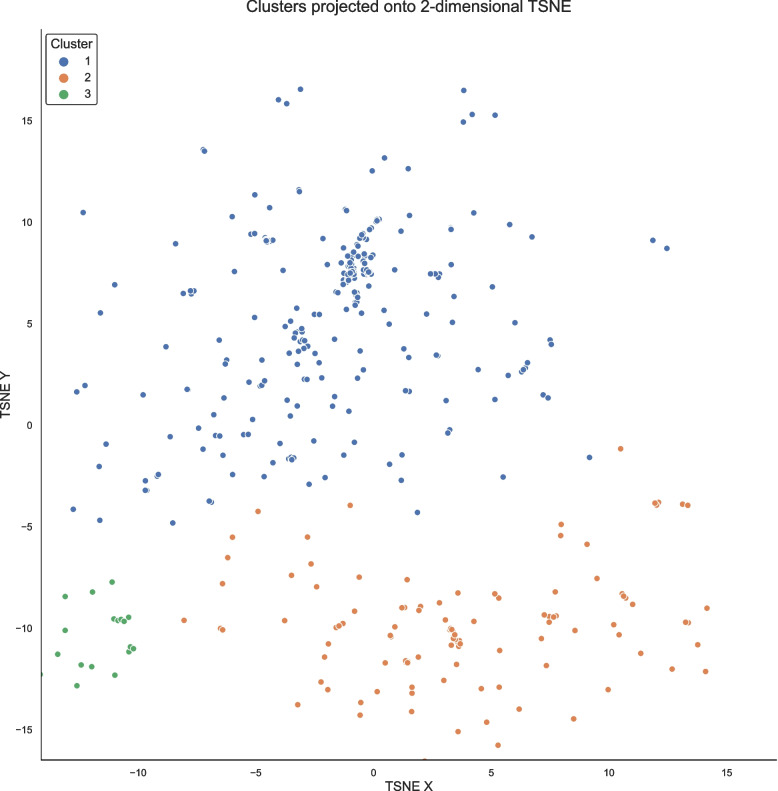
TSNE projection of longitudinal representations of acute illness presentations into two-dimensional manifold with designated clusters

Hierarchical clustering demonstrated three groups of 253, 123 and 38 participants in clusters 1, 2 and 3 respectively. Distinct clusters could be spatially visualised in two-dimensional space (Fig. 1). DECIDE datapoints could be downsampled to a comparable 2-dimensional space using functionable representations from DELPHIC TSNE downsampling (Additional file 2: Supplementary Fig. 1).

### Cluster interrogation

**Fig. 2:**
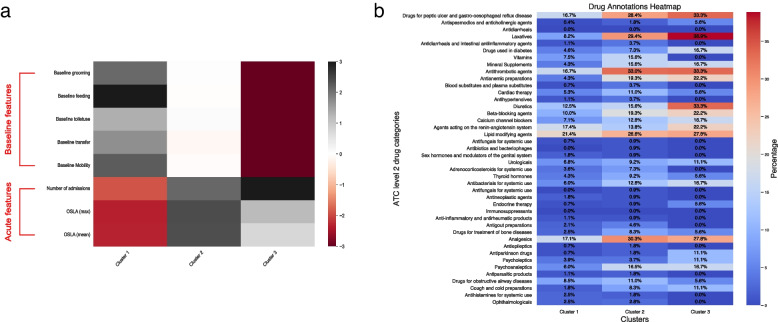
**a** Heatmaps of feature coefficient (left). **b** Prescribed medications percentage by cluster (right)

Within each cluster, related groups of variables, such as baseline cognition measures and summarised measures of acute illness physiology, were correlated and trended in an anticipated manner. Individual clusters revealed unique clinical differences in baseline cognition, frailty, delirium burden, physiological derangements and premorbid prescriptions (Fig. 2, full variables of each cluster in Additional file 4: Supplementary Fig. 2): cluster 1 was composed of participants with higher measures of baseline cognition (MMSE item scores, fewer years of education), lower premorbid frailty (lower clinical frailty score, higher Barthel Index), lower burden of delirium during acute illness (lower MDAS and OSLA scores), biochemically demonstrated lower C-reactive protein and creatinine. Participants in cluster 3 presented clinically orthogonally to cluster 1 (lower baseline cognition, higher frailty, more delirium, higher CRP and lower albumin). Participants in cluster 2 were clinically mid-range for baseline cognition, premorbid frailty, yet high in acute illness measures (delirium and arousal deficit scores, high CRP, high likelihood of physiological derangement). The specific summary parameter that correlated to a cluster subtype varied by individual biochemical or physiological feature: the standard deviation of CRP and AVPU were correlated to cluster 1 while for heart rate, the maximal readings are significant for cluster 2.

Participants in cluster 1 received lower number of prescriptions of cardiovascular medications (antithrombotics, anti-anginals, beta blockers, diuretics, lipid-lowering drugs), gastrointestinal medications (antacids and laxatives), required less mineral supplementation, less analgesia, as well as less likely to receive psychoanaleptics. Participants in cluster 3 demonstrated the highest prescription rates for all medications. There was no correlation between baseline cognition or frailty state with polypharmacy. Presence of analgesia and cardiovascular medications appeared most commonly in patients with polypharmacy.

### Cognitive decompensation prediction from clusters and longitudinally multi-modal data

**Fig. 3:**
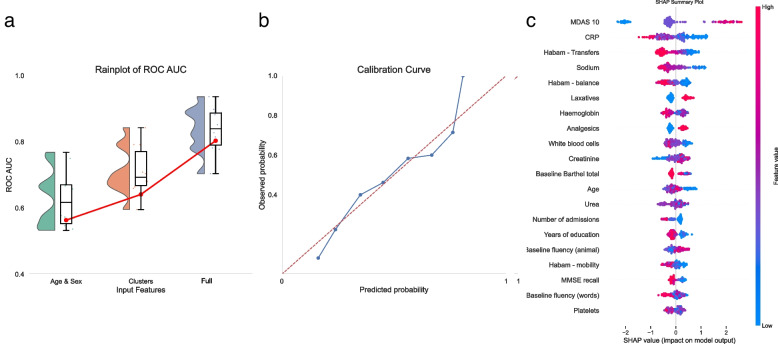
**a** Rainplots of training and testing hierarchical improvements in delirium in admission predictive performance with increasing model complexity; **b** calibration curve of high-dimensional model across range of predicted probability; **c** directional SHAPs map of predictive feature contributions to high-dimensional model

XGB prediction models using only age and sex achieved a relatively poor AUROC of 0.563 on the independent test dataset. Addition of cluster designation achieved a significant increase in performance with AUROC of 0.641 while the best performing model, incorporating all individual overlapping baseline and acute illness admission features, achieved AUROC of 0.797 (sensitivity 044, specificity 0.93, PPV 0.71, NPV 0.82) in an external test set (Fig. 3a). The best high-dimensional model demonstrated good calibration across the range of predicted and observed probabilities (Fig. 3b). SHAP values identified presence of pre-admission sleep–wake cycle disturbance (MDAS 10) as contributing most to predictive performance (Fig. 3c). Markers of underlying physiological, functional and cognitive function (hyponatraemia, low HABAM and total Barthel scores, lower number of years of education and poor baseline MMSE recall) plausibly related to underlying aetiologies, pre-existing prescriptions for constipation and pain relief were also predictive. Biochemical markers (CRP, platelets, haemoglobin and urea), all contributed information to prediction models but were bidirectional in their importance, suggesting complex non-linear relationships with delirium incidence.

### Long-term mortality prediction from clusters and longitudinally multi-modal data

**Fig. 4:**
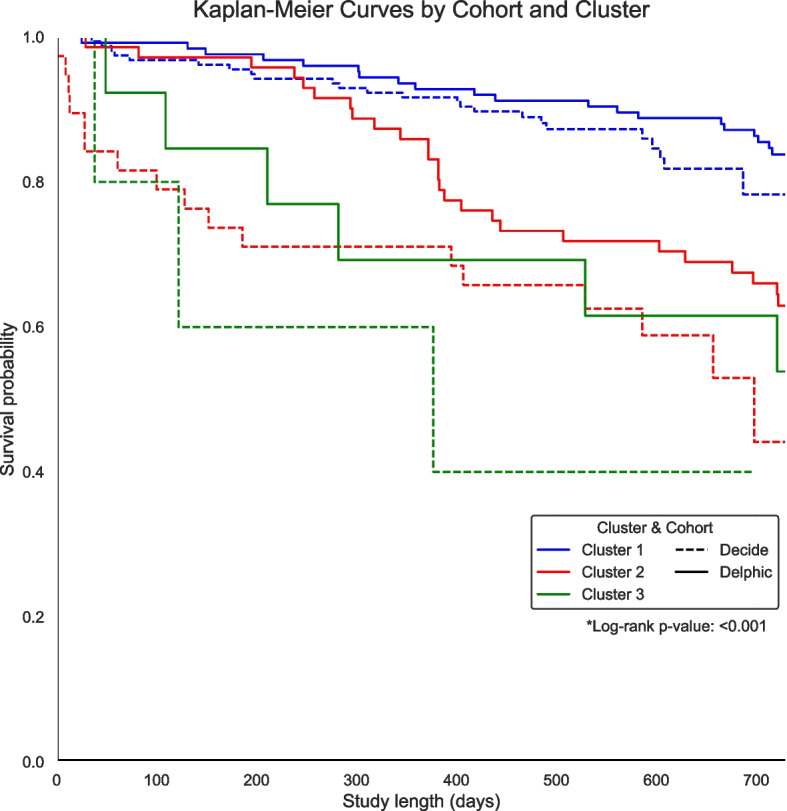
Kaplan–Meier plot of survival trajectories by cluster and cohort

Differences in survival trajectories were demonstrated on Kaplan–Meier plots (log-rank with cohort as interaction value, *p* value < 0.001, Fig. 4), with each analogous cluster in DELPHIC and DECIDE resulting in similar trajectories: participants in cluster 1 have the most and cluster 3 the least favourable survival trajectories respectively, with separation in trajectories appearing to initiate early from hospital discharge.

Predictive modelling for 2-year mortality demonstrated similar increasing performance with hierarchically more complex inputs. Baseline age and sex only produced a AUROC of 0.572, improving to 0.664 with clusters, and 0.690 (sensitivity 0.26, specificity 0.91, PPV 0.4, NPV 0.81) using full high-dimensional inputs. SHAPs for the full model identified fluctuations of clinical, physiological and biochemical inputs within an admission (haemoglobin, potassium, MDAS 5: *reduced ability to shift and maintain attention*, heart rate standard deviation) to contribute the most to prediction. Poorer baseline cognitive fluency (number of animals named) and fewer years of education were poor prognostic factors. Pharmacologically, prescription of “drugs for acid related disorders” was a marker of increased mortality risk while proinflammatory state, as demonstrated by increased white cell count and CRP, increases 2-year mortality risk (Additional file 5: Supplementary Fig. 3).

## Discussion

We applied unsupervised machine learning in two independent cohorts to demonstrate data-driven clusters within acute illness decompensation in older frailer patients. Clusters were prognostically associated with cognitive decompensation and 2-year mortality. In addition, we quantified relative fidelities of reduced and high-dimensional approaches to predict adverse outcomes, objectively demonstrating potential performance of high-dimensional, multi-modal and longitudinal prediction models. Taken together, our findings suggest different prognostic clusters are discernable, with implications for clinical management and inferring underlying disease mechanisms.

### Relative fidelities of deployable high-dimensional models and clinically informative low-dimensional clusters

Consistent clusters across independent cohorts confirm complex yet coherent longitudinal subtypes, and that binary diagnoses such as “delirium” encompass multiple individual conditions, each of which requires detailed ascertainment to maximise individualised care. Clusters offer an objective framework to more optimally direct patient care towards specific therapies most likely to benefit, while in clinical trials, prognostic clusters augment participant stratification.

Clusters were clinically informative beyond a description of age, sex and symptoms but the best predictive performances were produced by the full models, emphasising the added value of high-dimensionality when modelling combinations of baseline and acute features that frequently do not contribute linearly. For delirium within admission at first contact, high-dimensional models achieved standards suitable for clinical deployment. Our model, derived from an unselected inpatient sample across non-intensive care settings and externally validated, is unique. Other prediction models have been developed in post-operative or intensive care settings [21, 22], but tend to lack validation in a test partition.

### Evolution of delirium definition and acute illness description

Newly described clinical clusters suggest a need to evolve current classification approaches: acute cognitive decompensation and long-term mortality prediction requires robust inputs ascertained from both premorbid baseline and acute illness states. However, this inherently longitudinal approach challenges contemporary delirium constructs: the DSM5 conception of delirium is essentially cross-sectional, with references to premorbid baseline defined simply as a “*change from baseline attention and awareness*” [23].

Acute illness in older people is not well-captured by aggregated early warning scores [11]. Where physiological markers had large standard deviations, these were more prognostic than absolute measures. Moreover, the ranking of MDAS 5 (*reduced ability to shift and maintain attention*) suggest addition of specified cognitive domains, beyond “alert, voice, pain, unresponsive”, would offer easily implementable and feasible improvements on existing measures for highlighting acute illness severity.

Examining the features with the most prognostic information, there are mechanistic inferences that cannot simply be extended from younger cohorts. For example, while systemically proinflammatory states such as sepsis are associated with mortality in younger patients, this is not the case in our sample. The impact of polypharmacy emerges with more nuance in our models. Survival trajectories between cluster 1 and 3 diverged despite both groups receiving high prescription numbers, perhaps distinguishing subpopulations with appropriate versus inappropriate polypharmacy.

### Sleep–wake disturbance signifying a neurological substrate for delirium

The contribution of MDAS 10 (*disturbed sleep–wake cycle*) towards our delirium prediction model is a well-recognised association. Yet, it is unclear whether our finding represents a subacute sleep–wake cycle disturbance suggestive of an early pre or subsyndromal presentation of delirium and/or an association with a longer-term circadian disruption indicating an inherent risk factor. The implication of circadian circuits being a neuropathophysiological substrate of delirium is consistent with studies on brainstem connectivity during delirium. Abnormal functional connectivity in mesencephalic, posteromedial cortex and brainstem ascending reticular activity system networks [24] and abnormal resting state connectivity in suprachiasmatic nuclei nodes and connections [25] have been demonstrated on fMRI investigations of medical inpatients experiencing incident delirium. In addition, the hypothalamic–pituitary–adrenal axis, which is intimately related in anatomy and function to circadian rhythms, is abnormally activated with blunted cortisol output in patients who have experienced delirium [26, 27]. Isolation of relatively limited 10,000 suprachiasmatic nuclei neurones to be central neuropathophysiological substrates of delirium raises fundamental questions of what delirium is—does the phenotype simply reflect first decompensation of the most vulnerable part of complex brain networks? Is delirium a common final pathway of all cognitive dysfunctions, explaining why a spectrum of aetiologies can result in the same clinical syndrome, and when extended to known survival outcomes, therefore inherently simply a marker of cognitive frailty?

### Strengths and limitations

The principal advantage was the use of independent cohorts to test/train our models. Each cohort had comparable standards for outcome ascertainment and the models generalised well between samples despite some differences in baselines and acute illness presentations in the underlying cohorts.

Although we quantitatively derived clusters using distance-driven techniques, the process is dependent the number of modalities and dimensions employed. Pragmatically, the optimal number of subtypes is the number of distinct management options and prognoses, beyond which further separation has limited real-world impact. Second, while our TSNE projections are suggestive of clusters, we recognise caution in that global distance distortions may misrepresent true distances between data points when back-projected from low- to high-dimensional space (and vice versa). Third, our models utilised a limited number of modalities, namely clinical assessments and bloods, lacked socioeconomic markers such as index of multiple deprivation not already captured by educational attainment and baseline cognition, and include a relatively small sample size for each cohort. A greater number of investigative modalities and deeper phenotyping, contributing more distinct pathophysiological information, would allow deployment of more advanced deep learning approaches, maximising predictive performance and hence inferential power, for delirium nor long-term mortality, with the aim of identifying candidate pathophysiological targets for future therapies.

## Conclusions

In independent cohorts of older people with acute illness, we demonstrated structured subtypes using unsupervised machine learning. The clinical utility of these clusters and the further performance gain when deploying a high-dimensional approach are useful predictors of cognitive decompensation. Sleep–wake cycle disturbances may be central to cognitive decompensation. Our findings offer a proof-of-concept that clinical phenomenology in older frailer patients can be accurately characterised using data-driven approaches.

## Supplementary Information

Additional file 1. Supplementary Table 1 TSNE plot by cohort, demonstrating consistent representation of both dataset within the same two-dimensional projection.

Additional file 2. Supplementary Fig. 1 Prescribed medications defined by WHO ATC code level 2 by cohort.

Additional file 3. Supplementary Table 2 Final XG boost parameters for high-dimensional models after grid search tuning.

Additional file 4. Supplementary Fig. 2 Full heatmap of baseline and acute feature coefficients for each cluster.

Additional file 5. Supplementary Fig. 3 a) Rainplots of training and testing hierarchical improvements in 2-year mortality predictive performance with increasing model complexity; b) direction.

## Data Availability

Complete deidentified participant data for DELPHIC, along with study protocols, consent forms, and case report forms are available through the Dementias Platform UK Data Portal: https://portal.dementiasplatform.uk/. Data for DECIDE is available upon request to SJ Richardons and MP Gough, under the terms of the Creative Commons Attribution-NonCommercial 4.0 International (CC BY-NC 4.0) license, which allows for reuse and adaptation non-commercially, provided the original work is cited and changes are indicated.

## Abbreviations

AUROC: Area under receiver operating characteristic curve
CAMCOG: Cambridge Cognitive Examination
CFAS II: Cognitive Function and Ageing Study II
CRP: C-reactive protein
DECIDE: Delirium and Cognitive Impact in Dementia
DELPHIC: Delirium and Population Health Informatics Cohort
DSM5: Diagnostic and Statistical Manual of Mental Disorders, 5th Edition
HABAM: Hierarchical Assessment of Balance and Mobility
MDAS: Memorial Delirium Assessment Scale
MMSE: Mini-mental state examination
NEWS2: National early warning score 2
NPV: Negative predictive value
OSLA: Observational Scale of Level of Arousal
PPV: Positive predictive value
PRAUC: Precision recall receiver operating characteristic curve
SHAPs: SHapley Additive exPlanations
TICS-m: Modified Telephone Interview for Cognitive Status
TSNE: T-stochastic neighbour embedding
XGBoost: EXtreme Gradient Boosting
WHO ATC: World Health Organisation Anatomical Therapeutic Chemical classification
4AT: 4 “As” test
