# Supplementary material for: Clinical clusters during acute illness predict long-term mortality in older patients
Source: BMC Med. 2025 Dec 29;23:696. doi: 10.1186/s12916-025-04500-5 (PMC12752037; doi:10.1186/s12916-025-04500-5)
Supplement: Supplementary file 1 — Additional file 1. Supplementary Table 1 TSNE plot by cohort, demonstrating consistent representation of both dataset within the same two-dimensional projection. [file 12916_2025_4500_MOESM1_ESM.docx]

| **WHO ATC Drug** | **DELPHIC (%)** | **DECIDE (%)** |
| --- | --- | --- |
| A02 | 73.19 | 48.09 |
| A03 | 2.13 | 0.43 |
| A04 | 0.85 | 0.43 |
| A06 | 53.62 | 20.00 |
| C08 | 25.53 | 17.87 |
| C09 | 42.55 | 38.72 |
| C10 | 71.49 | 51.49 |
| D01 | 1.70 | 0.85 |
| D07 | 1.28 | 0.85 |
| G03 | 2.98 | 0.43 |
| G04 | 22.98 | 16.17 |
| H02 | 17.87 | 6.38 |
| H03 | 16.60 | 11.49 |
| J01 | 22.13 | 3.40 |
| J02 | 0.85 | 0.43 |
| L01 | 2.98 | 0.85 |
| L02 | 4.26 | 2.13 |
| L04 | 0.85 | 1.70 |
| M01 | 2.55 | 3.40 |
| M04 | 7.66 | 4.26 |
| M05 | 15.74 | 7.23 |
| N02 | 64.68 | 40.00 |
| N03 | 7.23 | 3.83 |
| N04 | 4.26 | 1.70 |
| N05 | 16.17 | 4.26 |
| N06 | 32.34 | 21.70 |
| P01 | 6.38 | 8.94 |
| R03 | 30.64 | 20.00 |
| R05 | 15.32 | 11.06 |
| R06 | 4.26 | 11.91 |
| S01 | 9.36 | 5.53 |

**Supplementary Table 1:** Prescribed medications defined by WHO ATC code level 2 by cohort
