# Supplementary figures and images for: Clinical clusters during acute illness predict long-term mortality in older patients
Source: BMC Med. 2025 Dec 29;23:696. doi: 10.1186/s12916-025-04500-5 (PMC12752037; doi:10.1186/s12916-025-04500-5)

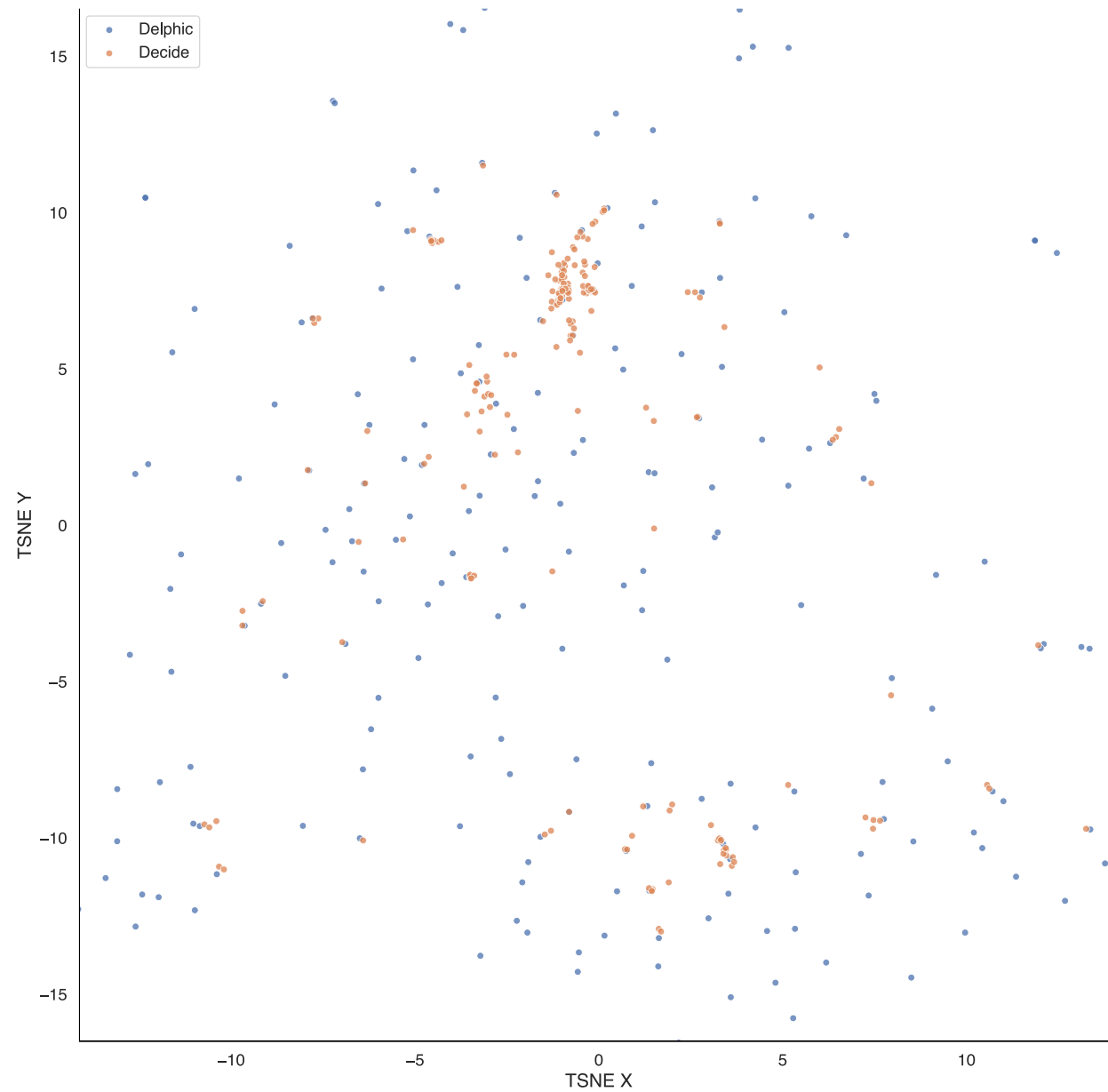

Supplement: Supplementary file 2 — Additional file 2. Supplementary Fig. 1 Prescribed medications defined by WHO ATC code level 2 by cohort. [file 12916_2025_4500_MOESM2_ESM.pdf]

Feature Coefficients By Cluster

Baseline features

Acute features

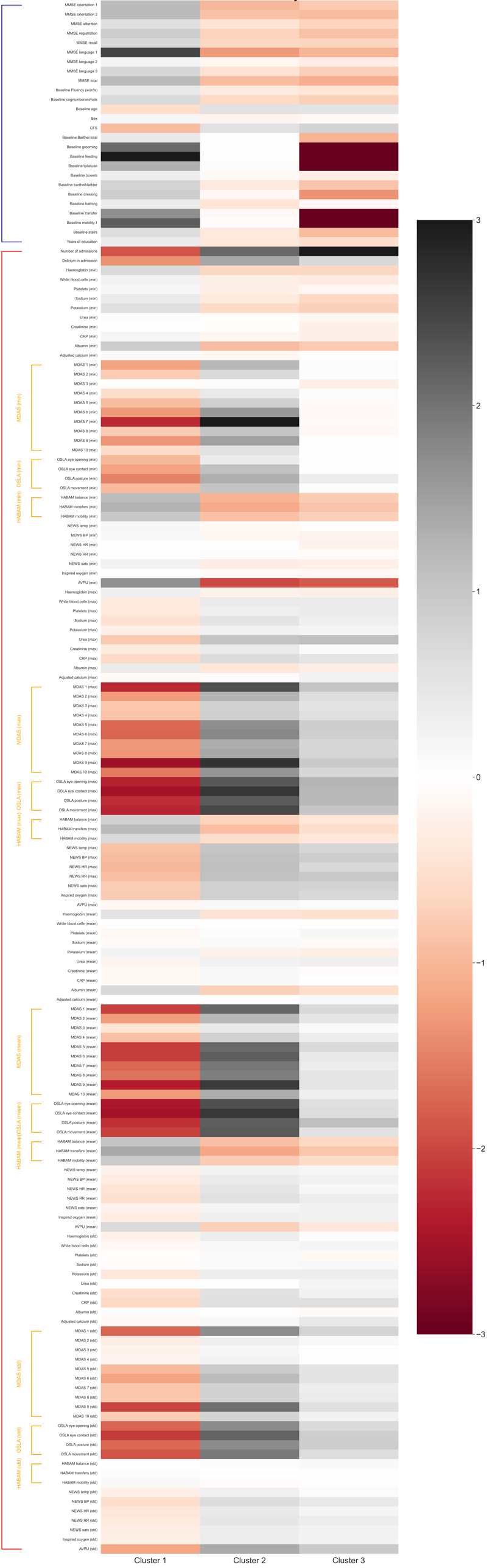

Supplement: Supplementary file 4 — Additional file 4. Supplementary Fig. 2 Full heatmap of baseline and acute feature coefficients for each cluster. [file 12916_2025_4500_MOESM4_ESM.pdf]

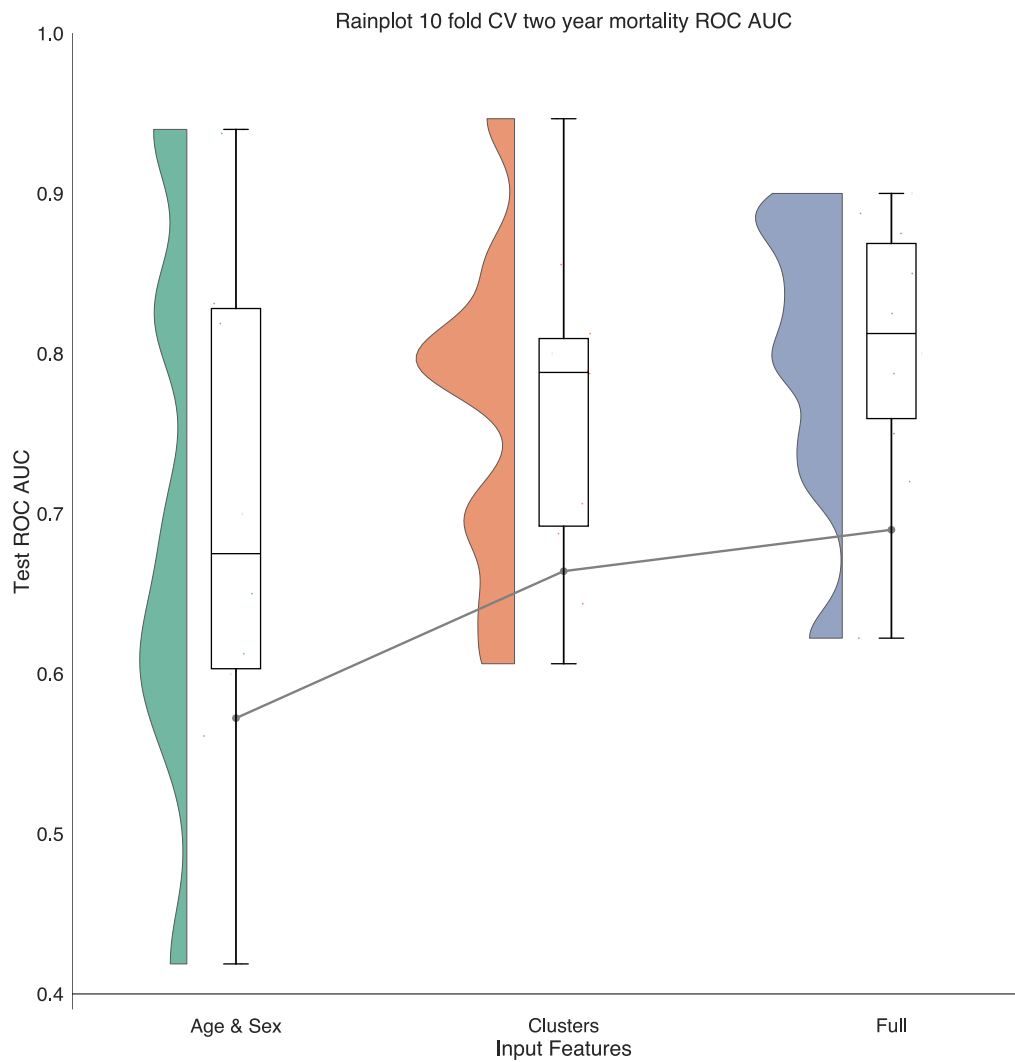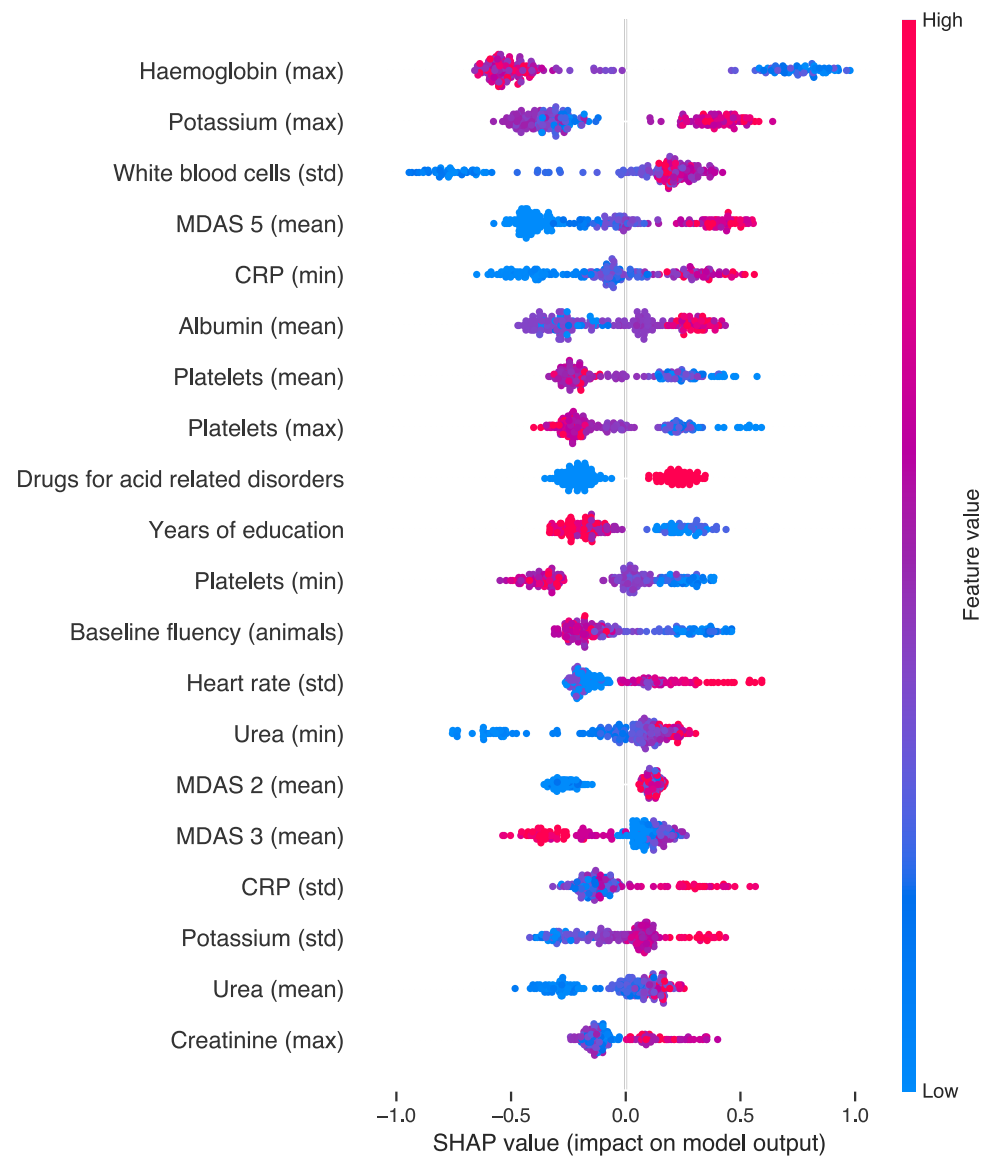

Supplement: Supplementary file 5 — Additional file 5. Supplementary Fig. 3 a) Rainplots of training and testing hierarchical improvements in 2-year mortality predictive performance with increasing model complexity; b) direction. [file 12916_2025_4500_MOESM5_ESM.pdf]
