# Supplementary material for: Clinical clusters during acute illness predict long-term mortality in older patients
Source: BMC Med. 2025 Dec 29;23:696. doi: 10.1186/s12916-025-04500-5 (PMC12752037; doi:10.1186/s12916-025-04500-5)
Supplement: Supplementary file 3 — Additional file 3. Supplementary Table 2 Final XG boost parameters for high-dimensional models after grid search tuning. [file 12916_2025_4500_MOESM3_ESM.docx]

|  | **Delirium in admission** | **Two-year mortality** |
| --- | --- | --- |
| **Learning_rate** | 0.1 | 0.1 |
| **Min child weight** | 0.9 | 0.9 |
| **Max depth** | 8 | 7 |
| **Gamma** | 0 | 0 |
| **Subsample** | 0.8 | 0.6 |
| **Column sample by tree** | 0.7 | 0.9 |

**Supplementary Table 2:** Final XG boost parameters for high-dimensional models after grid search tuning
